# Cerebral blood flow, autoregulation and vascular reactivity in normal pressure hydrocephalus: a systematic review

**DOI:** 10.1186/s12987-025-00720-2

**Published:** 2025-12-16

**Authors:** Afroditi Despoina Lalou, Zofia Helena Czosnyka, Marek Czosnyka, John Douglas Pickard

**Affiliations:** 1 Department of Clinical Neurosciences, Division of Neurosurgery, University of Cambridge & Cambridge University Hospital NHS Foundation Trust, Cambridge, England; 2 Department of Radiation Sciences, Umeå University, Umeå, Sweden; 3 Institute of Electronic Systems, Warsaw University of Technology, Warsaw, Poland

**Keywords:** Autoregulation, Cerebral autoregulation, Cerebrovascular reactivity, Cerebral blood flow, Hydrocephalus, Normal pressure hydrocephalus

## Abstract

**Background:**

Normal pressure hydrocephalus (NPH) is one of the few remediable causes of decline in gait and cognitive function in the ageing population. The roles of the cerebral circulation including haemodynamic reserve and cardiovascular co-morbidity in the pathogenesis, management and prognostication of NPH remain ill-defined. In this systematic review, we have updated Owler & Pickard’s review of 2001 to examine whether:

global/regional Cerebral Blood Flow (CBF) changes are specific to NPH, appropriately coupled to cerebral metabolism and/or reflect cerebral ischaemia; changes in global/regional CBF are predictive of outcome after CSF drainage; global and regional cerebrovascular autoregulation and reactivity are more sensitive predictors of outcome after CSF drainage than baseline regional CBF (rCBF); changes in global or regional CBF a cause or effect (‘chicken and the egg’) of NPH; a trial is warranted that combines assessments of haemodynamic reserve, CSF outflow resistance and response to temporary CSF drainage.

**Main body:**

We have systematically reviewed studies from 2000–2024 assessing CBF, autoregulation, and cerebrovascular reactivity (CVR) in adult NPH. Global and regional CBF were consistently reduced in NPH, particularly in periventricular white matter and deep grey matter, but these reductions were not predictive of shunt response. CVR varied from impaired to preserved and showed greater promise as a predictor of clinical improvement after CSF drainage or shunting. Methodological heterogeneity and small sample sizes limited meta-analysis. The interplay between CBF, CSF dynamics, and brain biomechanics remains complex, with evidence suggesting that impaired haemodynamic reserve may precede irreversible tissue damage.

**Conclusions:**

Baseline rCBF and borderline ischaemia reflect NPH networks but do not predict shunt response. CVR impairment does. It is suggested that randomised controlled trials be used to assess the predictive accuracy of combining the response of temporary CSF drainage with changes in CVR.

## Introduction

Reversible causes of dementia are crucially important to the individual, to their family and to society. It is almost 60 years since the symptoms of normal pressure hydrocephalus (‘Hakim’s triad’: gait disturbance, dementia and urinary incontinence with radiological hydrocephalus and normal baseline CSF pressure) and their response to CSF drainage were first described [1, 2] and yet there remain many uncertainties around the pathogenesis, diagnosis and management of Normal Pressure Hydrocephalus (NPH). The prevalence of symptomatic iNPH is of the order of 1.5% at the age of 70 rising to 7.7% at 86 years of age [3, 4] in parallel with the normal age-related increase in ventricular volume [5–7]. A further 3.7% at the age of 70 years have radiologically probable NPH, a proportion of whom progress to become symptomatic [8–10]. There remains a wide geographical disparity in the incidence of shunting for NPH [11].

Secondary NPH develops late after disease processes that are well known to obstruct the CSF circulation including, for example, subarachnoid and intraventricular haemorrhage, meningitis, traumatic brain injury, basilar artery ectasia or longstanding aqueduct stenosis. Such cases respond well to CSF diversion. By definition in idiopathic NPH, no such prior disease appears to have been present. A disturbance of the CSF circulation alone may not always be sufficient for developing the condition. Certainly, there is autopsy evidence of meningeal thickening, subependymal gliosis and periventricular white matter demyelination [12–14] and yet the relationship of CSF outflow resistance to post-drainage outcome, either temporary or permanent, is nonlinear [15]. MR studies have confirmed the presence of periventricular axonal stretch injury and, in some but not all studies, reduced volumes of the caudate, thalamus, putamen, pallidum, hippocampus and nucleus accumbens. [16–19] Reductions in volume of thalamus and caudate may also be part of normal ageing [8]. Co-morbidity and frailty are very common in iNPH [20–22]. There is a high incidence of vascular risk factors, cardio- or cerebro- vascular disease and white matter changes in many NPH subjects [23–30]. In iNPH with dementia, neuropathology often reveals evidence of Alzheimer change, cerebral small vessel disease (CSVD), multifocal infarcts, Parkinson’s disease or Parkinsonism (including Progressive Supranuclear Palsy, Corticobasal Degeneration and Multiple System Atrophy)[15, 31, 32]. CSF biomarker studies confirm that patients with iNPH and CSVD share common features of subcortical neuronal degeneration, demyelination, and astroglial response including the reduction in all APP-derived proteins [33]. Interestingly, it has been suggested that some cases of iNPH may have started with benign external hydrocephalus in infancy followed by deep white matter ischaemia in late adulthood [34, 35]. Certainly, head size is statistically larger in NPH patients compared with normal subjects [36, 37].

Hence, the cerebral vasculature may have a role in the pathogenesis of iNPH. However, such a role remains ill-defined, as does the ability of measurements of CBF and cerebrovascular autoregulation/reactivity to predict outcome after CSF diversion. The previous systematic review (2001 [38]) found that studies of CBF in iNPH had been inconsistent, not least in predicting the response to shunting. Studies had been hampered by heterogeneous patient groups of small size, incompatible definitions of clinical outcome, lack of longitudinal studies, and the resolving power of the then available technology to quantify periventricular regional CBF.

### Unresolved issues include:

- Whether there are changes in global and regional CBF that are specific to NPH and its clinical manifestations?
- Whether levels of global and regional CBF are appropriately coupled to cerebral metabolism and/or low enough to equate to ongoing cerebral ischaemia?
- Whether any changes in global or regional CBF are predictive of outcome after CSF drainage, both temporary and permanent?
- Whether global and regional cerebrovascular autoregulation and reactivity are more sensitive predictors of outcome and reversibility of symptoms in response to both temporary and permanent CSF drainage than baseline rCBF?
- Whether changes in whole brain CSF pressure-volume compensation and circulation, local tissue stress and loss of brain tissue volume relate to CBF and cerebrovascular autoregulation and reactivity? Whether any changes in global or regional CBF are the cause or effect (‘chicken and the egg’) of iNPH?

In this systematic review, we have updated the previous 2001 review and examined how far new knowledge has addressed these questions.

## Methods

From February 2020 up until June 2024, we performed a detailed search on Scopus, Cochrane, PubMed, and Web of Knowledge, using the key phrases “(((cerebral autoregulation) OR (cerebrovascular reactivity) OR (cerebrovascular resistance))) AND (normal pressure hydrocephalus)”. We set out the timeline to include manuscripts after 2000 up until 30/06/2024, due to the fact that Owler & Pickard [38] performed a systematic review of the relevant literature up to year 2000. The language in which papers were written did not matter, since we had access to the vast majority of the world languages. The articles had to be published - unpublished work or work awaiting publication was not considered. Case reports were excluded as they do not contribute to the questions asked nor to the need for adequate numbers of patients with NPH.

Articles had to involve idiopathic or secondary adult NPH patients with pre-shunting assessment of CBF or autoregulation at baseline and/or after CSF drainage or shunting. We compared all papers for consistency of references, background, analytical reporting of methods as well as reasoning and drawing of conclusions in an unbiased manner, taking into account NPH literature and its pathophysiological considerations. Cerebral Autoregulation was assessed separately to Cerebrovascular Reactivity (CVR).

The data from all the original articles was extracted using piloted data forms; the forms underwent some dynamic changes during the data extraction when new data or information arose. The RTI tool was used to assess bias in observational studies. The QUIPS tool was used for assessing risk of bias for study participation, prognostic factor measurement and outcome measurement.

The GRADE tool was used to classify the diagnostic and prognostic level of evidence as High, Moderate and Low [39]. A high level of evidence consisted of high quality studies with consistency and low to moderate (unclear) risk of bias; moderate level consisted of high quality evidence but inconsistent and with low to moderate risk of bias. Finally, low level of evidence included either high-moderate quality of evidence with inconsistency and high risk of bias, or moderate-low quality with at least inconsistency and/or high risk of bias. In order to uphold a standardised definition of Normal Pressure Hydrocephalus, the following criteria had to be met in defining and selecting the NPH group, as reported in Owler & Pickard [38]:

1. Full or incomplete clinical triad/primarily gait disorder;
2. ventricular dilatation on CT without significant atrophy;
3. absence of focal neurological deficit or focal pathology on CT;
4. normal ICP/CSF pressure (<15 mmHg) assessed using ICP monitoring or infusion study,
5. objective, well documented follow-up.

We reviewed and combined the findings prior to our 2001 review with the new research. This review design and protocol can be accessed in the PROSPERO register (registration number CRD42018090946).

## Results and discussion

**Fig. 1:**
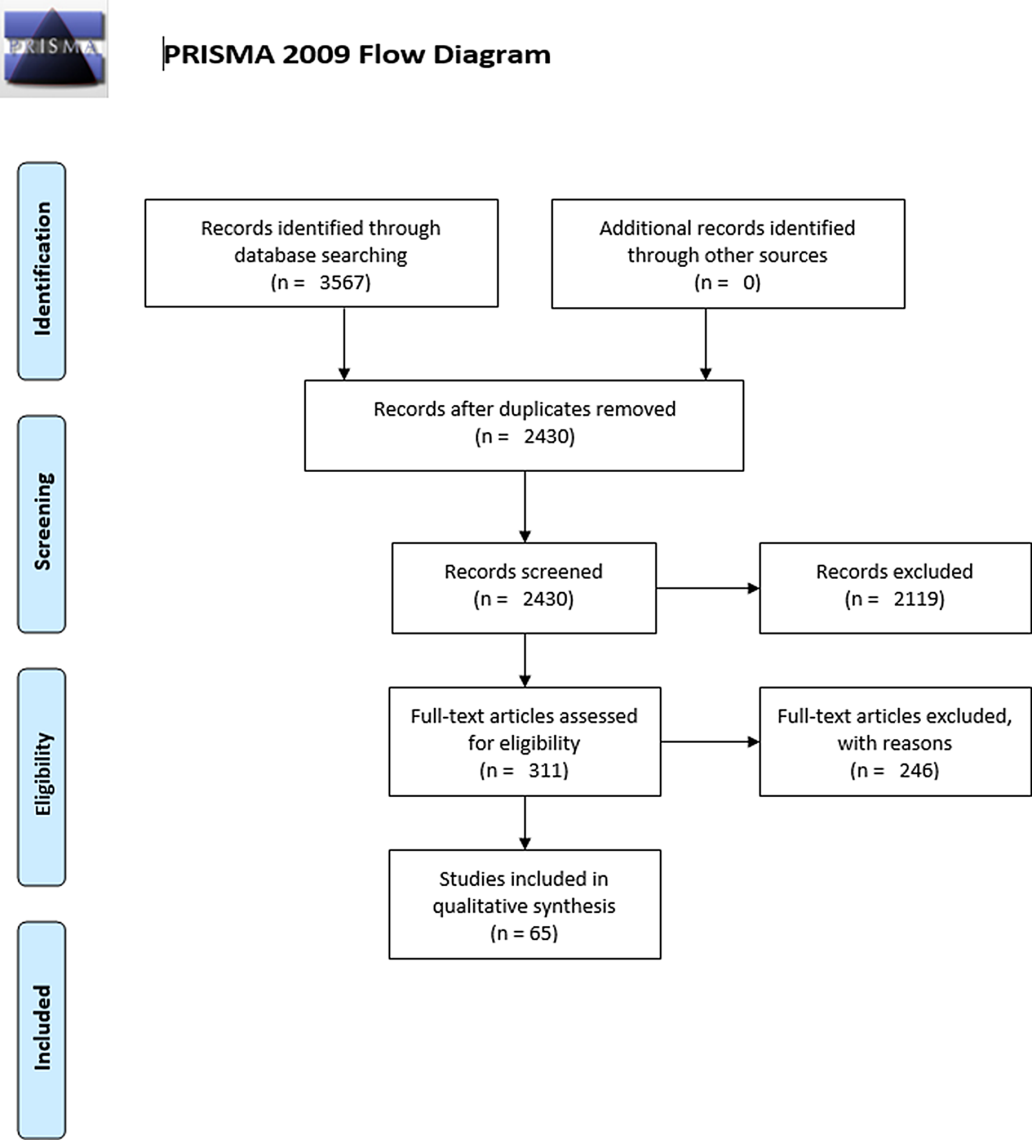
*From:* moher d, liverati a, Tetzlaff j, Altman DG, the PRISMA group (2009). Preferred reporting items for systematic reviws and metal-analyses: the PRISMA statement. PLoS med 6(7): e1000097. doi:10.1371/journal.Pmen1000097 for more information, visit http://www.prisma-statement.org

Figure 1 maps out the number of records identified from the searched databases, including those included and excluded with the reasons for their exclusions. All results reported refer to statistically significant results, unless stated otherwise. Our risk of bias assessment for observational and prognostic factor studies are presented in Supplementary tables 1 and 2.

### Overview

This review should be read in conjunction with Owler & Pickard’s review of 2001 [38] which matched the reports of global and regional CBF, autoregulation and reactivity to the methodology used. Unfortunately, very few studies since have included more than 50 patients and have not always included details of clinical presentation including duration of symptoms, or co-morbidity (frailty, Alzheimer’s, Parkinsons, cerebral small vessel disease (CSVD), systemic hypertension, diabetic status and other cardiovascular risk factors). It is now clear that there is much overlap in terms of patterns of CBF between CSVD and NPH. Few studies reported the scanning environment (eg quiet room, eyes closed) or what the patients were asked to think about during the CBF measurements. Such clinical and procedural heterogeneity renders statistical analysis challenging.

### Global and regional CBF (Table 1, Figure 2)

**Fig. 2:**
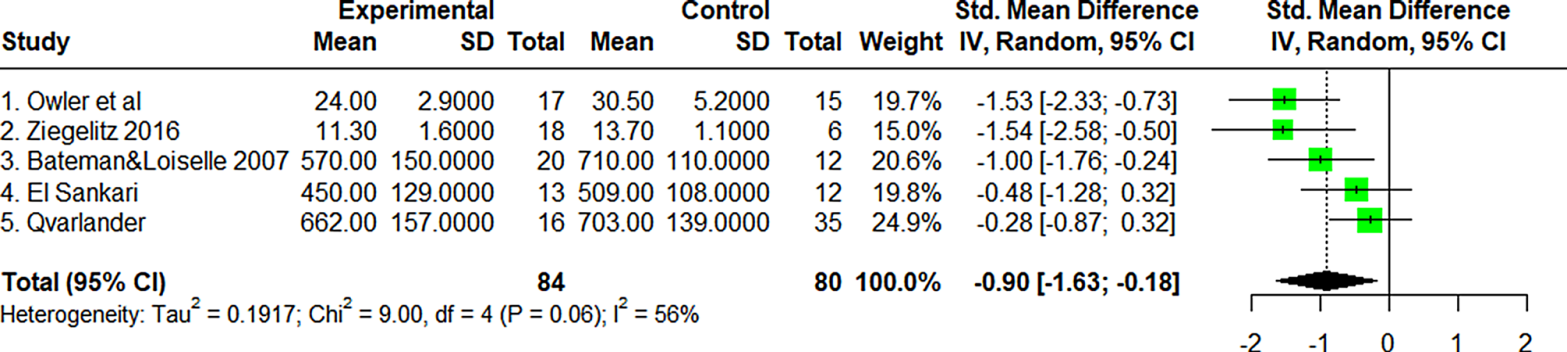
Global cerebral blood flow in normal pressure hydrocephalus versus age-matched controls at baseline (post-2001 studies). CBF values in mean and sd. Units are ml/(100g.Min) for studies 1,2 &3 and ml/min for the phase contrast (pc) MRI studies 4–6. *tau: estimated standard deviation of underlying effects across studies (Tau*^*2*^ *is only displayed in the random model). Chi*^*2*^ *(value of Chi-square for heterogeneity). Random: random analysis model (for meta-analysis). Standardised (std.) mean difference: calculated as Cohen’s delta, with Hedges’ bias correction. All studies have similar weight to the pooled estimate, as shown in the weight column and illustrated with the size of the green squares*

**Fig. 3:**
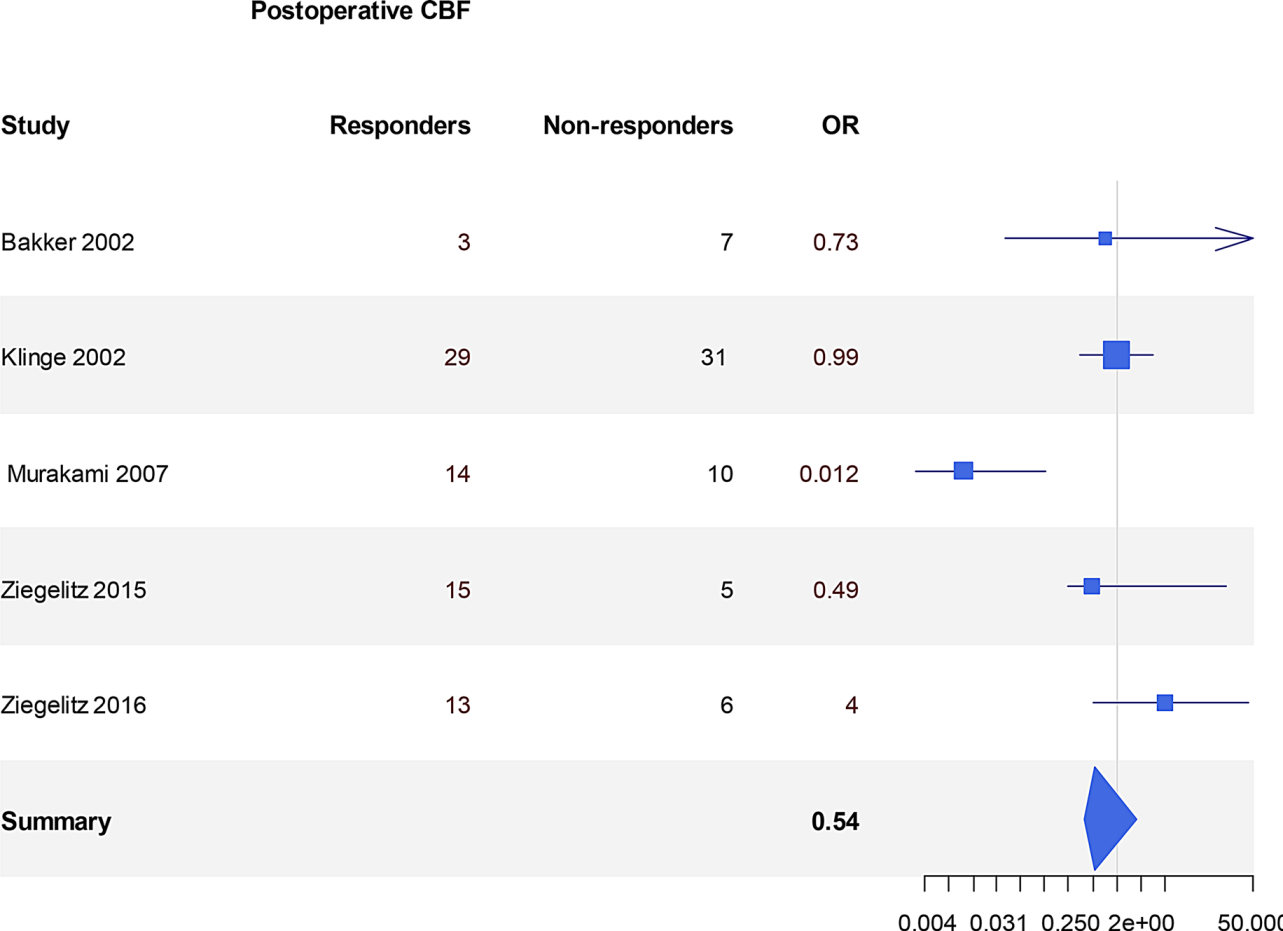
Global cerebral blood flow changes in improvers versus non-improvers. The odds ratio (or) represents whether shunting likely resulted in CBF increase (or >1) or no change/decrease (OR<1) in responders vs non-responders

**Fig. 4:**
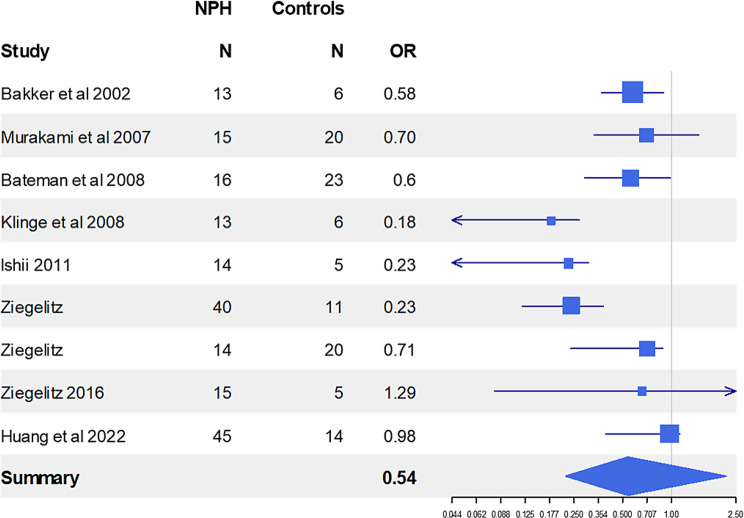
Predictive value of cerebral blood flow. The odds ratio (or) represents the probability of baseline CBF measurements to predict improvement after shunting and is based on the number of patients in each study

Overall, the 25 studies reported prior to 2001 provided Grade B evidence for reduced global CBF in 23 studies of 382 NPH patients with only 2 studies of 25 NPH patients showing unchanged global CBF compared to controls. PC MRI and perfusion MRI have been introduced since 2001. In Waldemar’s study [60], although global CBF was not reduced, the subcortical low flow area was enlarged. In the Qvarlander study [71], internal carotid but not vertebral artery flow was reduced. Post-2001 CBF measurements have confirmed that global CBF is reduced by 15.2% (sd 14.0%) in iNPH at baseline compared to controls (Figs. 2, 3 and 4).

**Table 1:** Global CBF studies at baseline: results of pre-2001 studies from owler & Pickard (re-graded using updated grade tool) combined with later studies

Global CBF					
Reference	Grade	Method	Number of NPH patients	Main findings	Comments
1. Greitz et al., 1969 (2 parts) [40, 41]	B	Intracarotid 133Xe clearance during Angiography	28 (21+7)	Reduced	In cases without a vascular component, correlation between ventricular dilation and ↓CBF
2. Salmon & Timperman et al., 1971a (2 parts) [42, 43]	C	As in 1 (Greitz et al)	12 (5+7)	Reduced	
4. Mathew et al., 1975 [44]	B	As in 1	15	Reduced	CBF unchanged between NPH&atrophy. No correlation between CBF &ventricular size
5. Hartmann et al., 1977 [45]	C	As in 1	11	Same	
6. Grubb et al., 1977 [46]	B	H_2_O^15^ PET Intracarotid	11	Reduced	CBF unchanged between NPH&atrophy. No definite pattern of CBF could be identified in NPH patients
7. Lying-Tunnell et al., 1977 & 1981 [47, 48]	C	AV-Difference N_2_O	7	Reduced	↓CBF especially in the most demented patients
8. Hayashi et al., 1984 [49]	B	As in 1	16	Reduced	↓CBF correlated with ↑ventricular size.
9. Kushner et al., 1984 [50]	B	As in 1	19	Reduced	No difference in CBF between NPH and non-NPH dementia patients
10. Meyer et al., 1984 [51]	B	133Xe inhalation	11	Reduced	
11. Meyer et al., 1985a [52]	C	133Xe inhalation & Xe contrast CT	8	Reduced	Frontal, temporal and parietal cortex
12. Meyer et al., 1985b [53]	B	Xe contrast CT	10	Reduced	
13. Brooks et al., 1986 [54]	C	C15O2 PET inhalation	3	Reduced	
14. Mamo et al., 1987 [55]	B	133Xe IV	25	Reduced	↓CBF compared to controls. No correlation between ventricular size and CBF reduction
15. Vorstrup et al., 1987 [56]	B	133Xe inhalation SPECT	17	Reduced	In 14/17 cases there was correlation between ↓CBF and ↑ ventricular size.
16. Graff-Radford et al., 1987 & 89 [24, 36]	C	133Xe inhalation SPECT	61 (26 +35)	Reduced	No difference between CBF in AD and NPH patients.
17. Meixenberger et al., 1989 [57]	C	133Xe inhalation	31	Reduced	Frontal cortex
18. Matsuda et al., 1990 [58]	B	133Xe inhalation	13	Reduced	↓CBF correlated with ventricular size.
19. Kimura et al., 1992 [59]	C	Xe contrast CT	7	Reduced	Generally ↓CBF in NPH
20. Waldemar et al., 1993 [60]	B	99m Tc-HMPAO SPECT & 133Xe inhalation SPECT	14	Same	No difference in tCBF.
21. Maeder et al., 1995 [61]	C	Xe contrast CT	4	Reduced	
22. Kristensen et al., 1996 [62]	B	99m Tc-HMPAO SPECT	31	Reduced	
23. Tanaka et al., 1997 [63]	A	Xe contrast CT	21	Reduced	
24. Klinge et al., 1998 [64]	B	H2O15 bolus PET	21	Reduced	Generally ↓CBF
25. Klinge et al., 1999 [65]	B	H2O15 bolus PET	10	Reduced	Generally ↓CBF
26. Owler et al 2004* [66]	B	H_2_^15^O PET	17	Reduced	
27. Bateman & Loiselle 2007 [67]	B	PC MRI	32	Reduced	Total blood inflow 20% ↓ Sagittal sinus outflow 35% ↓
28. El Sankari et al 2011 [68]	B	PC MRI	13	Reduced	Total blood inflow 12% ↓ (NS); Venous outflow unchanged (−2%; NS)
29. Yamada et al (2013)* [69]	B	SPECT	25	Reduced	Diffuse ↓ in global CBF pre-shunting similar to patients with ventriculomegaly due to age (no healthy controls).
30. Ziegelitz et al 2014 * [70]	B	Dynamic susceptibility contrast MRI	21	Reduced	Preoperative CBF ↓ in the global parenchyma deep GM
31. Qvarlander et al 2017 [71]	B	PC MRI	16	Same	↓Internal Carotid artery flow (Controls:519±123, NPH: 461±84ml/min,p=0.04)
32. Huang et al 2022 [72]	C	3D pulsed ASL MRI	32	Reduced	No healthy controls.

Xe: Xenon, Tc-HMPAO: Technetium – Hexamethylpropylene Amine Oxime. PC: Phase Contrast. ASL: Arterial Spin-Labelling

Various MR techniques (PC MRI, ASL MRI and DSC MRI), ^15^O PET and SPECT have now confirmed the older literature that global CBF is modestly reduced in NPH compared with age-matched healthy controls (Table 1 and Fig. 2).

#### Regional CBF and clinical correlations (Table 2)

With regard to regional CBF, the new consistent findings since 2001 are that, in addition to the frontal lobe, rCBF in the periventricular white matter and deep grey matter (thalamus and, more variably, caudate, hippocampus and lentiform nucleus) are reduced. One important methodological finding was that cerebellar CBF is modestly reduced which suggests that the use of the cerebellum as a control for SPECT studies is problematic. The reductions in rCBF in the deep WM and GM are in accord with reductions in volumes of these structures (but see below for discussion of the ‘chicken and egg’ conundrum) [86, 87].

**Table 2:** Regional cerebral blood flow at baseline including clinical correlations

Regional CBF					
Reference	Grade	Method	Number of NPH patients	Main findings	Comments
1. Mathew et al., 1975 [44]	**B**	Intracarotid 133Xe clearance during Angiography	**15**	Reduced	**Frontal lobe (grey & white matter) &** ACA territories (parietal lobe, corpus callosum, basal ganglia)
2. Meyer et al., 1985a [52]	**C**	**133Xe inhalation & Xe contrast CT**	**8**	Reduced	Frontal, temporal and parietal cortex, **thalamus** &fronto-temporal WM
3. Meyer et al., 1985b [73]	**B**	**Xe contrast CT**	**10**	Reduced	Frontal WM
4. Graff-Radford et al., 1987 & 89 [24, 36]	**C**	**133Xe inhalation SPECT**	**61 (26 +35)**	Reduced	**Frontal “region”**
5. Waldemar et al., 1993 [60]	B	99m Tc-HMPAO SPECT & 133Xe inhalation SPECT	14	Same *	*Slight ↓ in the centrum semiovale. *NPH patients had lower frontal/parietal ratio CBF
6. Maeder et al., 1995 [61]	**C**	**Xe contrast CT**	**4**	Reduced	Frontal cortex & white matter
7. Kristensen et al., 1996 [62]	B	99m Tc-HMPAO SPECT	31	Reduced	Inferior frontal &temporal cortex. frontal & parietal WM
8. Owler et al (2004a) [74]^*^	B	H2^15^O PET &3T MRI	17	Reduced	Cerebellum, **thalamus**, head of caudate and putamen.
9. Momjian et al (2004) [75]	B	H_2_^15^O PET & 3TMRI	12	Reduced	Paraventricular borderzone regions. WM, with abnormal gradient from lateral ventricles towards the subcortical WM**(PVWM).**
10. Sasaki et al (2007) [76]	B	SPECT (voxel based analysis)	30	Reduced	Corpus callosum. Either frontal dominant or diffuse pattern. Medial &lateral in urinary incontinence
11. Klinge et al (2008) [77]	B	H_2_^15^O PET uptake	68	Reduced	Greater impairment correlated with reduced tracer uptake in mesial frontal and anterior temporal areas.
12. Yoon et al (2009) [78]	B	SPECT	10	Reduced	bilateral **thalami**, anterior and posterior cingulate gyri, right prefrontal area, right caudate nucleus and left parahippocampal gyrus
13. Takaya et al (2010) [79]	B	SPECT	14	Reduced	parietal lobe, **lateral &medial frontal cortex**, lateral temporal &occipital cortex, cingulate gyrus, precuneus, frontal WM, semioval center,corpus callosum; caudate, **thalamus**, pons, cerebellum
14. Ishii et al 2011 [80]	A	N-isopropyl-p-^123^I iodoamphetamine SPECT. Relative CBF in each voxel was calculated by normalizing each voxel activity to the global brain activity.	84	Variable patterns: frontal perfusion predominantly reduced. The medial and lateral frontal, parietal, and occipital CBFs relatively increased at high convexity.	Multicentre study in 26 centres; basal ganglia and thalamus not analysed. CBF in the peri-sylvian and ventricular areas, where the cerebrospinal fluid spaces are dilated, is also reduced – partial volume issue.
14. Ziegelitz et al 2014 [70]	B	Dynamic susceptibility contrast MRI	21	Reduced	basal medial frontal cortex, hippocampus, lentiform nucleus, **PVWM** & central GM
15. Virhammar et al (2017) [81]	B	pCASL perfusion MRI	21	Reduced	**PVWM,** cerebellum and pons lentiform nucleus & **thalamus**
16. Takahashi et al (2019) [82]	B	CBF SPECT & MRI	30	Reduced	↑rCBF in the lower part of the high convexity area. ↓rCBF in medial frontal lobes, left lateral frontal lobe, and left parietotemporal region
17. Azuma et al (2019) [83]	B	^123^ I-IMP SPECT MRI	39 (15 - iNPH/AD+ 24 - iNPH/AD−)	The putamen was the only region in which rCBF was significantly lower in iNPH/AD− patients than in iNPH/AD+ patients	No controls No significant correlation between gait and rCBF in any region.
18. Agerskov et al (2020)* [84]	B	DSC MR perfusion	20	Same	ROIs in upper mesencephalon and lower pons only.
19. Huang et al 2022 [72]	B	3D pulsed ASL MRI	32	Reduced	high convexity, temporal lobe, precuneus, and **thalamus.**
20. Kang et al 2023 [85]	B	^18^F-florbetaben (E-FBB) PET FDG PET MRI	39	Mixed patterns	↑rCBF in the high convexity of frontal and parietal cortical regions ↓rCBF in ventrolateral frontal cortex, supramarginal gyrus and temporal cortical regions.

*Owler et al (2004) & Momjian et al (2004) analysed the same cohort of patients using different voxel analyses

All the post-2001 studies that have investigated the frontal cortex confirmed the pre-2001 findings of frontal hypoperfusion but with less consistent findings in the lateral and medial frontal cortices and frontal white matter. Studies that included the periventricular white matter, thalamus, basal ganglia and cerebellum as ROIs demonstrated hypoperfusion. There were also reports of hypoperfusion of the cingulate gyrus, corpus callosum, pons and temporo-parietal areas.

Frontal hypoperfusion (lateral, medial and basal) accords with the loss of both executive and motor function in NPH. Cognitive functions depend upon efficient functioning of distributed brain networks connected by white matter tracts. In the case of CSVD, such frontal hypoperfusion has been related to a regional predominance of WMH and white matter tract disruption in the frontal lobes, consistent with cortical disconnection, that would provide a plausible structural basis for selective loss of executive function [28, 88–91]. Regarding neurocognitive symptoms, impaired wakefulness was associated with decreased rCBF in the anterior cingulate gyrus and anterior periventricular white matter in two out of the three studies of neurocognitive results.

Of the 12 studies on rCBF, 4 described the relationship between gait and rCBF. Whilst different regions were investigated, the thalamus as well as frontal and periventricular structures demonstrated a correlation with gait impairment in 3 studies. In contrast, Azuma’s study [83] did not find any significant correlation between gait and rCBF in any regions. White matter surrounding the lateral ventricles, especially in the frontal lobes, is key to gait function. Fibres in these areas connect brain regions likely involved in iNPH, such as the supplementary motor cortex, basal ganglia, and thalamus, and in elderly people without iNPH, WM hyperintensities in the frontal lobe and periventricular WM have the strongest relationship with impairments in balance and gait [92, 93].

Akai et al [12] reported that the most prominent post-mortem finding in NPH was demyelination of the white matter supplied by the anterior and middle cerebral arteries. While the peripheral arcuate region of the white matter region was reasonably maintained, the deep white matter demonstrated a marked reduction in the number of myelinated axons. The number of axons themselves was also reduced. Whether this is a cause or effect of reduced blood flow in periventricular regions is not clear. Such changes may also have distant effects due to deafferentation and thereby reduction in CBF in those regions affected. Such functional deafferentation is consistent with recent studies of Resting State Networks [73, 94–96].

Axons may only need to be stretched across the dilated ventricles to influence functioning. As early as 1947 Yakovlev had proposed that gait disorder was due to stretching of axons responsible for gait [97]. Of course, periventricular stretch and compression impacts on blood vessels as well as axons (see below [98–101]).

There is some intriguing disagreement over whether rCBF is *increased* in the high convexity area (Table 2). Such increases are counter-intuitive and yet have also been identified in longitudinal studies of normal aging without dementia where extensive bilateral regions showed greater relative increases in rCBF in the group with progressive WM abnormalities compared with the stable WM group over time. Kraut et al [102] suggested that ‘these increases in rCBF associated with WM pathology might relate to changes in resting cerebral cortical activity (and thus metabolic requirements), as accommodations are made for less efficient interregional WM connections. That is, regions of brain that are interconnected by deficient WM would have to maintain activity for longer periods or at a higher level to communicate with one another even in the resting state, either through the deteriorating pathways that had been used before WM degeneration had begun, or through less efficient indirect pathways. This prolonged cortical neural activity would require increased blood flow to meet the increased metabolic demands.” The authors accepted that this idea runs counter to several studies of the relationship between cerebral glucose metabolism and WM abnormalities, where in general it has been found that increased severity of WM disease correlates with reduced regional rates of cerebral glucose metabolism.

### Evidence for and against borderline cerebral ischaemia (Table 3)

It is a matter of some debate whether changes in the cerebral circulation are a cause or effect of NPH. There is strong evidence of decreased vascular density in ageing animals including humans [126–129]. As noted above, cerebrovascular hypertensive changes are common in NPH. White matter hyperintensities are very common in NPH [28, 29]. The normal-appearing white matter surrounding white matter hyperintensities is associated with decreased structural integrity and perfusion and increased risk of their growth [130, 131]. However, CBF reduction may also be the consequence of ventricular dilatation, reduced metabolism and other secondary changes. Unfortunately, there have been no longitudinal studies comparing the time course of changes in rCBF with tissue volume nor of comparative recovery after shunting. However, it is well established that, at least in MCI, hypoperfusion may be dissociated from atrophy [86, 87].

**Table 3:** Results of cerebral metabolism studied either separately or simultaneously with cerebral blood flow

Reference	Grade	Method	Significant findings Baseline	Significant post-shunt changes	Comments
**Cerebral oxygen consumption**					
1. Grubb 1977 [46]	C	Intracarotid ^15^ O-oxyhaemoglobin C^15^O & H_2_^15^O PET	CBF & CMRO_2_ were both reduced in 6 NPH patients compared with controls	No relevant data	No WM measurements and no OEF data.
2. Lying-Tunnell 1981 [47]	C	Kety-Schmidt N_2_O	↓CBF (−33%) ↓CMRGlu ↓oxygen uptake	All parameters improved	
3. Brooks 1986 [54]	B	^15^O PET	↓CBF ↓CMRO_2_ OEF no change	No change	Cortical CBF only reported; only CMRO_2_ changes were significant
4. Ishikawa 1989 [103]	C	Continuous inhalation of 15O-labelled CO2 and O2 gases in PET scanner	Reduced CBF and CMRO2 in lower cortical slices particularly frontal with a diffuse increase in OEF. No WM measurements.	No data.	Post-SAH patients: 6 NPH and 7 non-NPH; no healthy controls.
5. Miyamoto 2007a/b [104, 105]	C	^15^O & C^15^O PET	No significant differences in rCBV and rOEF between iNPH (N=9) and healthy age-matched controls with asymptomatic ventriculomegaly (N=10).	↑CMRO_2_ and no change in rOEF in frontal WM, putamen and thalamus in good responders (5); no change in CMRO2 and reduced rOER in the same regions in poor responders (N=3).	
6. Zhuang 2023 [106]	B	3D multi- gradient echo to assess ventricular CSF volumes with OEF mapping (MRI QQ-CCTV algorithm); ASL	Significant negative correlation between OEF and normalised ventricular volume, cortical and deep GM.	No clinical data. No WM data. No significant finding with CBF or CMRO_2_.	QQ-CCTV and ASL are complex techniques. A fall in OEF would suggest true ischaemia rather than oligemia but CBF would be expected to be low which was not found ^105,106^.
**Cerebral glucose metabolism**					
7. Jagust 1985 [107]	C	^18^F-FDG PET	Global hypometabolism in 3 patients		2 Idio; 1 post SAH No CMRGlu calculation
8. George 1986 [108]	C	^18^F-FDG PET		3 chronic HC patients: ↑CMRGlu post-shunt	
9. Kaye 1990 [109]	C	^18^F-FDG PET	Global ↓CMRGlu	Early ↑CMRGlu with return to near normal by 2 years with maintained clinical improvement	Case report
10. Tedeschi 1995 [110]	B	^18^F-FDG PET	Global hypometabolism – very heterogenous patterns		18 patients; 7 improved post-shunt; frontal biopsy: heterogeneous findings
11. Calcagni 2013 [111]	C	^18^F-FDG PET		CMRGlu increased in all cortical regions post shunt; no correlation with symptoms; no specific regional variation	N=20
12. Townley 2018 [112]	B	^18^F-FDG PET Co registered MRI - partial volume correction	No specific pattern of significant cortical hypometabolism. Significant striatal hypometabolism.		N=7; healthy controls
13. Miyazaki 2019 [113]	A	^18^F-FDG PET Cerebellar cortex used for ratio calculation	↓frontal and temporal ratios in prodromal and iNPH. ↓ratio in thalamus and striatum in iNPH		12 iNPH 33 with DESH only 32 asymptomatic with ventriculomegly and DESH
14. Chiaravalloti 2020 [114]	B	^18^F-FDG PET		Significant ↑ left frontal and parietal regions; ↓right frontal	10 patients No CMRGlu analysis
**Intracerebral microdialysis**					
15. Agren-Wilsson 2003 [115]	B	Right frontal 10mm catheter; 0-7mm from the frontal horn Brain tissue oxygen tension PtiO_2_ Pre and 2–4 hours post CSF infusion study and drainage to zero CSF pressure		Lactate and pyruvate increased; no change in Lac/Pyr ratio PtiO_2_ increased in 5 of 8 patients (~ 3 mmHg)	10 patients
16. Eide 2010 [116]		Left frontal (20mm catheter) extending from cortical surface to 20mms below; controls using normal frontal cortex.	Modest changes: ↑Lac (63% of patients) ↓Pyr (60% of Patients) ↑L/P: no change (8% of patients) ↑Glut (38% of patients)	Modest changes during Extended lumbar drainage ↓Lac (29% of patients) ↑Pyr (82% of patients) ↓L/P (43% of patients) ↓Glut (75% of patients)	No differences in baseline or during EDT between responders and non-responders.
**Magnetic Resonance Spectroscopy**					
17. Kizu et al 2001 [117]		^1^H-CSI	intraventricular lactate peaks in NPH (n=9) not in controls or Alzheimer’s/Picks.		
18. Braun 2003 [118]	C	^1^H MRS – ratio analysis	WM; NAA/Cr no change		18 adults <40 years, 14 >40. WM lateral to lateral ventricle
19. Shiino 2004 [119]	B	^1^H MRS – ratio analysis	No controls	High NAA/Cr – favourable outcome	Secondary NPH only; WM lateral to lateral ventricle
20. Kubas 2006 [120]	B	^1^H MRS – ratio analysis	NAA/Cr ↓8%	Not done	Left frontal VOI
21. Del Mar Matarin 2007 [121]	B	^1^H MRS – ratio analysis		NAA/Cr ↑ 6%	No controls Medial frontal
22. Lenfeldt 2008a [122]	A	^1^H MRS – ratio analysis	NAA/Cr ↓13%	Higher in improved patients	Frontal WM ELD for 3 days
23. Algin 2010 [123]	B	^1^H MRS – ratio analysis	NAA/CR ↓11% NAA/Cho ↓13%	No change; no correlation with outcome (n=18)	Frontal lobe
24. Lundin 2011/2013 [124, 125]	A	^1^H MRS – absolute quantification	Thalamus – NAA ↓10% Frontal deep WM – Cho ↓7.9%	Thalamus: No change Frontal deep WM: Cho ↑4.5%	

The use of positron emission tomography (PET) in cerebrovascular disorders has greatly improved our understanding of the clinical pathophysiology of cerebral ischaemia [132, 133]. The measurement of regional cerebral blood flow (CBF), oxygen consumption (CMRO_2_), oxygen extraction (OEF) and blood volume (CBV) has permitted the identification of three successive stages of severity which are characterized by (1) an isolated rise of CBV (reflecting a vasodilatation that largely underlies the mechanism of CBF autoregulation); (2) a moderate fall in CBF with normal CMRO_2_ and increased OEF (a fully compensated stage defining oligaemia); (3) a depression of CMRO_2_ as CBF falls further (denoting true ischaemia).

In NPH, both cerebral oxygen and glucose metabolism were reduced to a similar degree to CBF with no change in oxygen extraction fraction (OEF) [47, 54, 104, 105]. The majority of studies reported reduced glucose metabolism in the frontal region, thalamus, caudate and subcortical periventricular white matter [106–114]. Hence, the reduction in global/cortical CBF was coupled to the reduction in oxidative brain metabolism and therefore there was no evidence of significant cerebral ischaemia. Despite this preservation of OEF, there is some evidence of low-grade, covert/impending/borderline/chronic ischaemia/misery perfusion in NPH (Table 3). Despite significant, modest baseline changes in lactate (increase) and pyruvate (decrease), there was no significant change in lactate/pyruvate ratio [116]. These changes do not meet the strict microdialysis definition of cerebral ischaemia - increased L/P ratio with reduced pyruvate [134]. However, extended lumbar drainage resulted in modest falls in lactate, increase in pyruvate and fall in L/P ratio in some patients [115]. Neither baseline values nor changes with ELD differed between responders and non-responders. Brain tissue oxygen tension increased modestly (by 3mmHg) in 5 of 8 patients after the combination of a constant pressure CSF infusion study and CSF drainage down to zero pressure [115].

Magnetic Resonance Spectroscopy studies of N-Acetylaspartate/Creatine (NAA/Cr) ratio in the frontal lobe have demonstrated no change or falls of 8–13% which increased after shunting [117–125]. Absolute quantification of NAA revealed a significant fall in NAA in the thalamus (↓10%) and in choline, but not NAA, in the deep frontal WM (↓7.9%). Thalamic NAA did not normalise after shunting [124]. Low NAA may be an indicator of neuronal loss that may be reversible. Changes in NAA are not an early indicator of cerebral ischaemia. Reduction in NAA occurs more slowly than increases in lactate after focal ischaemia and is greater in the core of an infarct than in peripheral areas [135]. The thalamic findings are in accord with the reduction in thalamic volume in NPH.

### Effect of CSF drainage (tap test and shunting) on global and regional CBF (Tables 4–5)

Overall, global CBF assessed using TCD, ^15^O PET, PC MRI and ASL MRI does not appear to increase after spinal tap test or shunting (Tables 4 and 5). Interestingly, one study using DSC MRI in 20 subjects did report an increase in global perfusion. Surprisingly, there have been no reports of combining ELD with global CBF studies.

**Table 4:** CBF before and after temporary CSF withdrawal

Ref	Grade	**NPH I and/or 2**^**0**^	_	Method	Main Findings	Comments and correlations
Owler et al 2002		-	Summary of previous evidence	Systematic review of literature	↑ in CBF equally likely as ↓after CSF withdrawal	
1. Mori et al (2002) [136]	A	22(I3 2° post SAH; 9 I)	Regional CBF pre & 10 minutes post CSF drainage in shunt responders vs non-responders (no controls; prospective)	CSF removal (30–50 mls) [137]; I]IMP SPECT with arterial sampling and arterial blood gas/MAP monitoring	Baseline clinical characteristics & CBF values were not significantly different between responders (15) & non-responders (7). 10 Minutes post-drainage, CBF significantly more in all regions in responders (101±39%) than in non-responders (46 ± 40%).	> 80% increase in CBF after CSF removal was predictive of response to shunt surgery with 77% accuracy. Corrected CBF change of <20% indicated nonresponse to shunting (see Table X).
2. Hertel, Walter et al (2003) [138]	B	27(I)	Correlation of preoperative STT with CBF/CBV changes and response to subsequent shunt (retrospective)	STT (>40mls); ^99m^Tc -bicisate SPECT (normalisation to cerebellum); pwMRI (Gad)	33% of patients showed clinical and regional perfusion improvement post-STT; 33% showed increases in regional perfusion but no post-STT improvement; 33% showed no clinical improvement post-STT and no increases in cerebral perfusion. There were no differences between regions.	It was not possible to distinguish white matter from grey matter on SPECT. Both groups who showed increases in post-STT cerebral perfusion variably improved post shunt. The group who were imaging negative and did not improve post-STT clinically were not shunted (see Table X).
3. Walter, Hertel et al (2005) [139]	B	28(I) 9 patients common with Hertel, Walter et al 2003.	Correlation of preoperative STT with CBF/CBV changes and response to subsequent shunt (prospective)	STT (>40mls); Pw -MRI (Gad) pre and 24 hours post-STT (with analysis by visual inspection)	7/28 patients: rCBV and gait improved post-STT (all improved post-shunt). 9/28 patients: rCBV but not gait improved post-STT (7/9 shunted and improved to some degree). 12/28 patients: no improvement in either rCBV or gait post-STT (none of this group were shunted).	Improved brain perfusion after STT is more sensitive than clinical assessment alone in predicting improvement after a shunt (see Table 5).
4. Dumarey et al (2005) [140]	B	40(I&2° x 8)	Regional CBF & gait measurements pre & post STT (retrospective)	STT (>30mls); ^99m^Tc HMPAO SPECT (with SPM analysis)	No significant difference between pre- and post-STT SPECT images. Gait improvement after STT was associated with ↑rCBF in the middle frontal gyrus & left parahippocampal gyrus.	Only 14 patients shunted. No data on WM v GM.
5. Virhammar et al (2014) [141]	A	20(I)	rCBF before & 30mins, 4hrs and 24 hrs after STT; comparison with gait improvement after STT (prospective)	STT; pCASL-MRI	Overall, no significant increase in rCBF in any region after CSF removal compared with baseline. In patients with ↑ CBF in the periventricular lateral and frontal white matter after the CSF STT, gait function improved more than in patients with↓ CBF in these regions.

**Table 5:** Global and regional CBF before & after shunting including correlation with outcome after shunting

Reference	Grade	Method	No of shunted patients	Main findings	Comments	Post-CBF correlation to improvement
**Global CBF**						
1. Greitz et al, 1969 [142]	C	Intracarotid 133Xe clearance during Angiography	7	Increase		No
2. Salmon & Timperman et al., 1971b [43]	C	As in 1.	12	Increase	Grey matter more than white matter. Variable change in white matter	NA
3. Lying-Tunnell et al., 1977 & 1981 [47, 48]	C	AV-Difference N_2_O	7	Increase	Temporary	NA
4. Kushner et al., 1984 [50]	B	As in 1.	19	Increase		No
5. Meyer et al., 1984 [51]	C	133Xe inhalation	10	Increase	Inconclusive	Inconclusive
6. Brooks et al., 1986 [54]	C	C15O2 PET inhalation	8	No change		NA
7. Mamo et al., 1987 [55]	B	133Xe IV	25	Increase	No region or pattern. Also temporary vs sustained	No
8. Vorstrup et al., 1987 [56]	B	133Xe inhalation, SPECT	17	No change	No pattern	No
9. Graff-Radford et al., 1987 [143]	C	133Xe inhalation, SPECT	30	No change	No region or pattern	No
10. Meixensberger et al., 1989 [57]	C	133Xe inhalation		No change	Frontal regions	NA
11. Matsuda et al., 1990 [144]	B	133Xe inhalation	13	No change		NA
12. Klinge et al., 1998 [64]	B	H2O15 bolus PET	21	No change		NA
13. Klinge et al., 1999 [65]	B	H2O15 bolus PET	10	No change		NA
14. Matsuda et al., 1999 [145]	B	Xe contrast CT	16	Increase	No pattern	yes
15. Bakker et al 34(2002) [146]	C	TCD	10	No change		NA
16. Klinge et al (2002) [147]	B	15-O-H2O PET	59	No change	Same patients, both global and regional studies (see below)	No
17. Bateman & Loiselle (2007) [67]	C	MRI (1.5T) with flow sequences	32	No change		No
18. Ziegelitz et al (2016) [148]	B	Dynamic susceptibility contrast MRI	20	Increase		Yes
19. Virhammar et al (2020) [149]	B	pCASL perfusion MRI	18	No change	Potential methodological limitation w ASL	No
**Regional CBF**						
20. Meyer et al., 1985a [51]	C	133Xe inhalation	10	Increase	Cortices, basal ganglia, **frontal WM**	Inconclusive
21. Meyer et al., 1985b [52]	B	133Xe inhalation	7	Increase	Cortices, basal ganglia, **frontal WM**	Yes
22. Waldemar et al., 1993 [60]	B	99m Tc-HMPAO SPECT & 133Xe inhalation	13	Increase	Subcortical structures, some cortical	Yes
23. Kimura et al., 1992 [59]	C	Xe contrast CT	7 post SAH	rCBF returned to within normal limits in the.	white matter of the frontal and temporo-parieto-occipital lobes	CBF restoration closely correlated with clinical improvement and reduction in ventricular dilation and periventricular lucency.
24. Shimoda et al., 1994 [150]	B	Xe contrast CT	22	Increase	Some subcortical, not basal ganglia	Yes
25. Maeder et al., 1995 [61]	C	Xe contrast CT	2	Increase	Frontal cortex and WM	NA
26. Tanaka et al., 1997 [63]	B	Xe contrast CT	21	Increase	WM more than grey	Yes
27. Klinge et al (2002) [151]	B	15-O-H2O PET	11	Increase	Frontal inferior, frontal rostral, temporal inferior, temporal dorsal, cingulum, parietal	yes
28. Mataró et al (2003) [152]	C	HMPAO-SPECT with SPM analysis	15	Increase	prefrontal dorsolateral areas, frontal premotor, medial prefrontal, **frontal WM,** inferior parietal lobule & basal ganglia	NA
29. Tullberg et al (2004) [153]	A	HMPAO SPECT – relative regional CBF (rrCBF) using cerebellum as the reference	28 (16 with impaired wakefulness)	Increase	thalamic, frontal and hippocampal grey matter.	Yes (impaired awareness only, no other symptom studied)
30. Murakami et al (2007) [154]	B	IMP SPECT + MRI with 3D-SSP	24	Increase	frontal base and the anterior part of limbic areas.	NA
31. Klinge et al (2008) [155]	B	15-O-H2O PET	47	Increase	superior mesial frontal areas	Yes
32. Ishii et al (2011) [80]	A	IMP SPECT rCBF relative to global brain activity.	84	Increase	3 different patterns of reduction in rCBF identified (anterior, posterior and mixed)	NA
33. Ziegelitz et al (2014) [70]	C	DSC MRI with Gd-DTPA using the occipital lobe as reference	20	Increase	PVWM	Yes
34. Ziegelitz et al (2015) [156]	B	DSC MRI with Gd-DTPA	20	Increase	hippocampus, PVWM, and in the cingulum, thalamus & basal ganglia	Yes
35. Nocun et al 2015 [157]	B	HMPAO SPECT	16	Increase 3–6 days post-shunt	Variable between patients – predominantly in frontal lobes	NA
36. Ziegelitz et al (2016) [148]	C	CT perfusion	17	CBF in responders increased postoperatively in all anatomical regions by 2.5–32%;	Significant increase in caudate head, normal appearing WM and periventricular WM.	Postoperative perfusion in the periventricular WM regions showed positive correlations with the gait score, continence score and total iNPH scale score.
37. Tuniz et al (2017) [158]	B	Diffusion and DSC MRI (3T) with gadobutrol using the occipital lobe as reference	13	Increase	basal ganglia and PVWM area.	Yes .
38. Azuma et al (2019) [83]	C	IMP SPECT MRI	39	Increase	putamen, amygdala, hippocampus, and parahippocampal gyrus. White matter not analysed (not sure why)	No
39. Agerskov et al (2020)* [84]	B	DSC MR perfusion	20	Increase	Mesencephalon & pons ↓rCBF in the mesencephalon of non-responders	Yes
40. Huang et al 2022 [72]	B	3D pulsed arterial-spin labelling (PASL) MRI	32	No change	Very weak correlations	No

Most studies have confirmed that rCBF increases after shunting in the frontal lobe, periventricular white matter and deep grey matter. Importantly, there is some evidence that such increases correlate with gait improvement after shunting, even when there has been no clinical improvement after a spinal tap test. In a landmark study, the Umea group have demonstrated enhanced activity in the SMA accompanying improved finger motor performance after extended CSF drainage consistent with changes in subcortical connections [159]. In hydrocephalus, periventricular stretch of axons is probably greatest frontally. In NPH, such axonal stretch may be compounded by CSVD but, unlike CVSD, is more likely to be reversible after shunting [17, 98, 101]. However, functional improvement is not associated with restoration of ventricular size. Lenfeldt et al surmised that CSF withdrawal “improved neuronal operational ability, possibly by reversing a subcortical chronic ischaemia related to cerebrovascular disease in periventricular pathways to and from SMA [24–26]. This would result in improved signalling in these circuits, normalizing the motor planning process, giving further support for the view of INPH as a hypokinetic condition caused by malfunction in cortico-basal ganglia-thalamo-cortical circuits with special involvement of frontal areas”. This notion is also supported by correlations between motor function and blood flow changes in frontal subcortical regions after shunting, and deficiencies in neuronal integrity in the same area [77].

#### Cerebrovascular reactivity and autoregulation before and after shunting (Tables 6 & 7)

Studies of CVR and Autoregulation might be expected to be more sensitive than baseline measurements of CBF by quantifying haemodynamic reserve, both global and regional [176–179]. Global CVR to either ACZ or hypercapnia was generally reduced in NPH patients and increased after shunting (Tables 6 & 7). This reduction in cerebrovascular reactivity is consistent with the arterioles being already maximally dilated as a result of local ischaemia, particularly in the white matter. Interestingly, such maximal arteriolar dilatation is not reflected in significant increases in cerebral blood volume [44, 46, 54, 103]. This lack of a significant increase in CBV may be because the cerebral resistance arteriolar blood volume is only a relatively minor component of global CBV. Veins and capillaries form the major component of global CBV and are minimally responsive to changes in cerebral perfusion pressure or hypercapnia [180, 181]. The interpretation of the responses to changes in CO2 and Diamox are more straightforward, albeit complicated by changes in MABP with hypercapnia. Both hypercapnia and Diamox, but not hypocapnia, induce significant increases in both CBF and CBV [182–184]. Unfortunately, there are no published studies of CVR before and after temporary CSF drainage.

**Table 6:** Cerebrovascular reactivity & autoregulation: baseline

Reference	Grade	Method	No of Patients	Main findings	Comments
**Cerebrovascular reactivity**					
1. Hartmann et al., 1977 [45]	B	CO2	11	Impaired CVR	
2. Meyer et al., 1984 [51]	C	CO2	7	Impaired CVR	
3. Lee et al., 1998 [160]	B	CO2	11	Impaired CVR	
4. Klinge et al 1999, 2002a [65, 162]	C/B	^15^O-H_2_O PET; ACZ	10 of 33	Impaired CVR	Baseline CVR lower in responders than non-responders (NS)
5. Chang et al., 2000 [163]	B	ACZ	41	Impaired CVR	
6. Chang et al (2003) [164]	**B**	^99m^Tc HMPAO & ACZ	48	Impaired CVR	↓CVR in 30 responders compared to normals. 4 non-responders with↓ CBF but preserved CVR.
7. Jarus-Dziedzic et al (2005) [165]	C	TCD; ACZ	13	Preserved CVR (not different to controls)	CVR better in NPH compared to atrophy.
					↓ CVR in atrophic group (n=10) vs controls
8. Yamada et al (2013) [69]	B	^99m^Tc-ECD SPECT +ACZ	25	Impaired CVR	
**Autoregulation**					
9. Mathew et al., 1975 [44]	**B**	Intracarotid 133Xe clearance during Angiography	15 (10 2;5)	rCBF and rCBV increased after CSF removal (?volume)	The greater the increase in CBF in the upper frontal and postcentral areas after CSF removal, the better the post-shunting outcome.
10. Schmidt et al., 1990a [166]	B	Kety-Schmidt Captopril and CO2	14	Preserved autoregulation	
11. Czosnyka et al (2002) [167]	B	TCD (Mx) & CSF infusion test; CT/MRI	35	Preserved global autoregulation	Correlation with Rout. Autoregulation tended to be worse in patients with ischaemic changes on CT/MRI
12. Owler et al (2004b) [168]	B	H_2_^15^O PET and MRI; CSF infusion study; finite element analysis	15	With increases in CSF pressure, global CBF was reduced. Regional CBF decreased in the thalamus and basal ganglia, and in white matter regions in proximity to the ventricles.	
13. Momjian et al (2004) [75]	B	H_2_^15^O PET and MRI T1&T2-weighted; CSF infusion study.	12	White matter CBF reduced in NPH compared with controls with an abnormal gradient from the lateral ventricles towards the subcortical WM. Reduction in CPP and raised CSF pressure reduce CBF which is maximal in the paraventricular watershed region.	
14. Czosnyka et al (2005) [169]	B	CSF infusion test & non-invasive MAP	68	Preserved global autoregulation	Better global autoregulation associated with high Rout.
15. Lalou et al (2018) [170]	B	CSF infusion test (PRx) & non-invasive MAP	131	Preserved global autoregulation	Better autoregulation correlated with increased Rout

^**^ *No Healthy controls. Patients with NPH symptoms that were not selected for shunting were used as comparison, together with CSF dynamics parameters*

CVR: cerebrovascular reactivity, TCD: Transcranial Doppler, Mx:Mean Flow velocity Index, PRx: Pressure Reactivity Index, MAP: Mean arterial Blood Pressure, CPP: Cerebral perfusion Pressure

**Table 7:** Cerebrovascular reactivity pre and post shunt including correlation with outcome after shunting

Ref	GRADE	Method	No of patients	Main findings	Post-shunt CVR: correlation with improvement
1. Meyer et al 1984 [51]	C	Xe-CT+5%CO2 +100%O2	7	Global increase	yes
2. Lee et al., 1998 [160]	C	TCD response to 5% CO2;	11	Global increase	NA
3. Klinge et al., 1999 [65]	B	15-O-H2O PET + ACZ	10	Global increase	yes
4. Miyake et al,1999 [161]	B	ACZ; ICP and XeCT	41 (15 iNPH)	Preoperatively, ICP increased after ACZ administration significantly more in the shunt-effective group than in the non-effective group. The percentage increase in CBF was similar in the two groups.	NA
5. Chang et al., 2000 [163]	B	99mTc-HMPAO +ACZ	41	Global increase	NA
6. Bakker et al (2002) [146]	C	TCD response to 5% CO2;	10	Global increase	NA
7. Klinge et al (2002a-d) [162, 171–173]	A	^15^O-H_2_O PET; ACZ	53	Global increase in responders at 7d & 7m; in both high and low vascular risk groups; No increase in CVR in non-responders.	Yes. Increase in global CVR at 7d correlated with improved gait; Increase in global CVR at 7m correlated with improvement in visual attention and verbal memory
8. Chang et al (2003) [164]	B	tec99m HCMP & ACZ	48	Global increase	yes
9. Chang et al (2003, 2009) [174, 175]	B	SPECT and Xe-CT, ACZ	162	Global increase	yes
10. Yamada et al (2013) [69]	B	SPECT +ACZ	25 NPH 30 controls	Global decrease pre-shunt compared with controls that improved post-shunt	yes

In contrast, very few studies of cerebral autoregulation have been reported and all recent studies are from one centre using CSF infusion studies to manipulate cerebral perfusion pressure. With increases in CSF pressure, there was a variable increase in arterial blood pressure between individuals and global CBF was reduced. This change in arterial blood pressure, with more modest changes in CSF pressure than the conventional end-stage Cushing response, has subsequently been attributed to the existence of an intracranial baroreceptor [185–187]. Crucially, during the plateau of an CSF infusion study, CBF decreased in the basal ganglia, thalamus and periventricular WM. The strength of cerebral autoregulation reached a minimum in the white matter close to the periventricular watershed area.

The integrity of AR also depends on cardiovascular comorbidity and abnormalities within the cerebral mantle. In Lalou’s study [188], the relationship between the global autoregulation pressure reactivity index (PRx) and the profile of disturbed CSF circulation and pressure-volume compensation in relation to outcome after surgery was examined. The PRx was negatively correlated with resistance to CSF outflow: patients with normal CSF circulation tended to have worse autoregulation. Given the importance of co-morbidities to the diagnosis and outcome of shunting in iNPH, it is important to note that AR is intact in Alzheimer’s disease but variably impaired in CSVD [189, 190].

Surprisingly, there are no reported studies of autoregulation before and after temporary CSF drainage or shunting.

### Prediction of outcome: baseline global & regional CBF, cerebrovascular reactivity and autoregulation (Tables 8 & 9, Figures 5 & 6)

None of the post-2001 studies have related global or regional CBF to outcome. Such measurements prior to 2001 were not entirely consistent in their findings with no strong evidence for using global or regional CBF before surgery as a useful prognostic factor for shunt response. The studies reporting the role of baseline CBF in predicting outcome post shunting were not based on secure statistical power analyses and the number of patients recruited overall was small (average of 20–30 patients with only 2 studies including >50 patients). There was grade C evidence of the predictive value of CBF in NPH. 5 studies of 89 patients’ global CBF showed different thresholds for positive and negative predictive value of CBF. The other 5 studies of 99 patients showed no correlation. rCBF was investigated in 3 studies or 82 patients, 2 of which reported that the ratio of the anterior and posterior CBF was high in improvers (70 patients total). Overall, there was very little evidence that either global CBF or the pattern of rCBF at baseline was correlated with outcome post shunting.

**Fig. 5:**
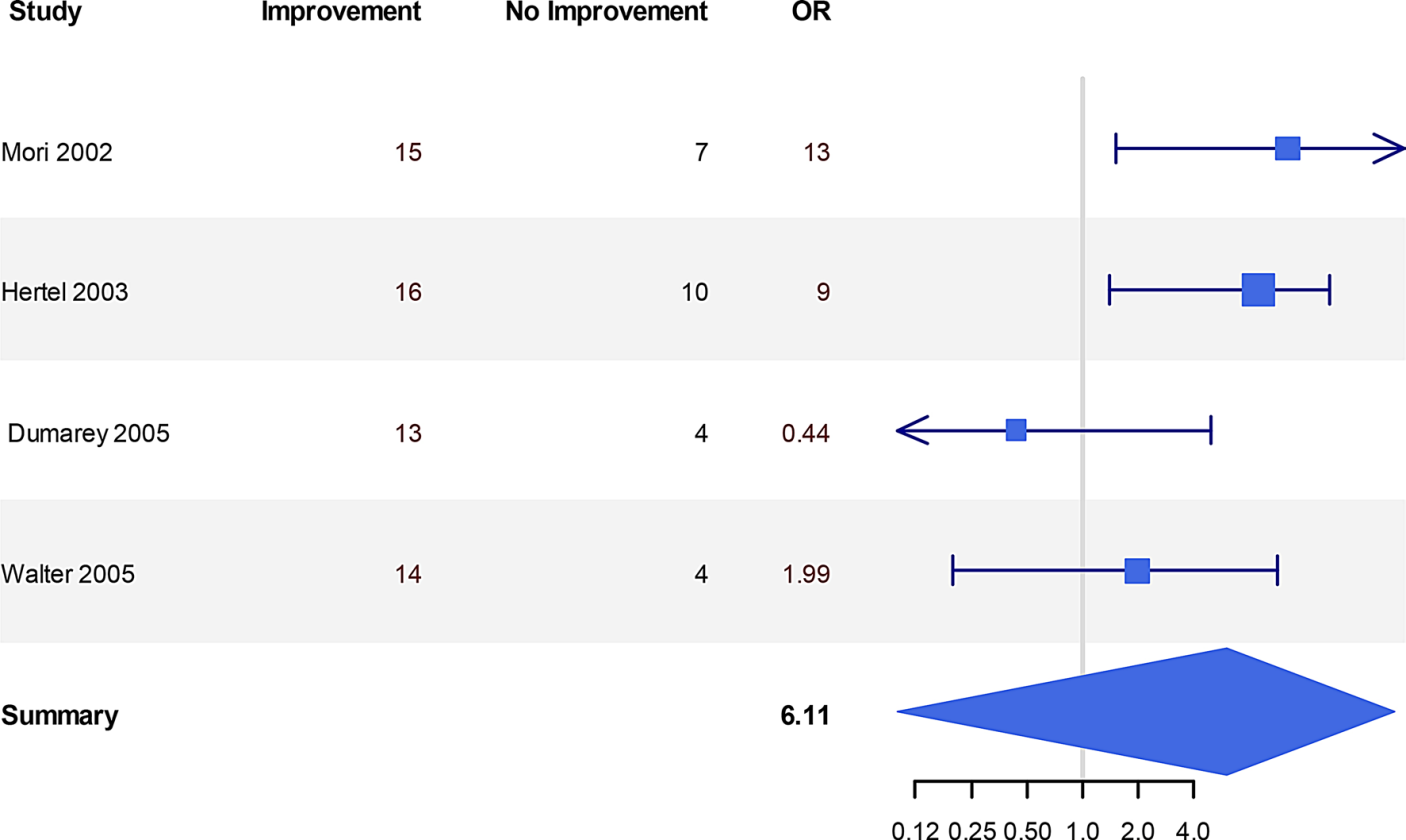
Predictive value of changes in CBF before and after temporary CSF withdrawal (spinal tap test -STT). The odds ratio (or) reflects the number of patients in whom an increase in CBF after a spinal tap test predicted improvement after shunting or not

**Fig. 6:**
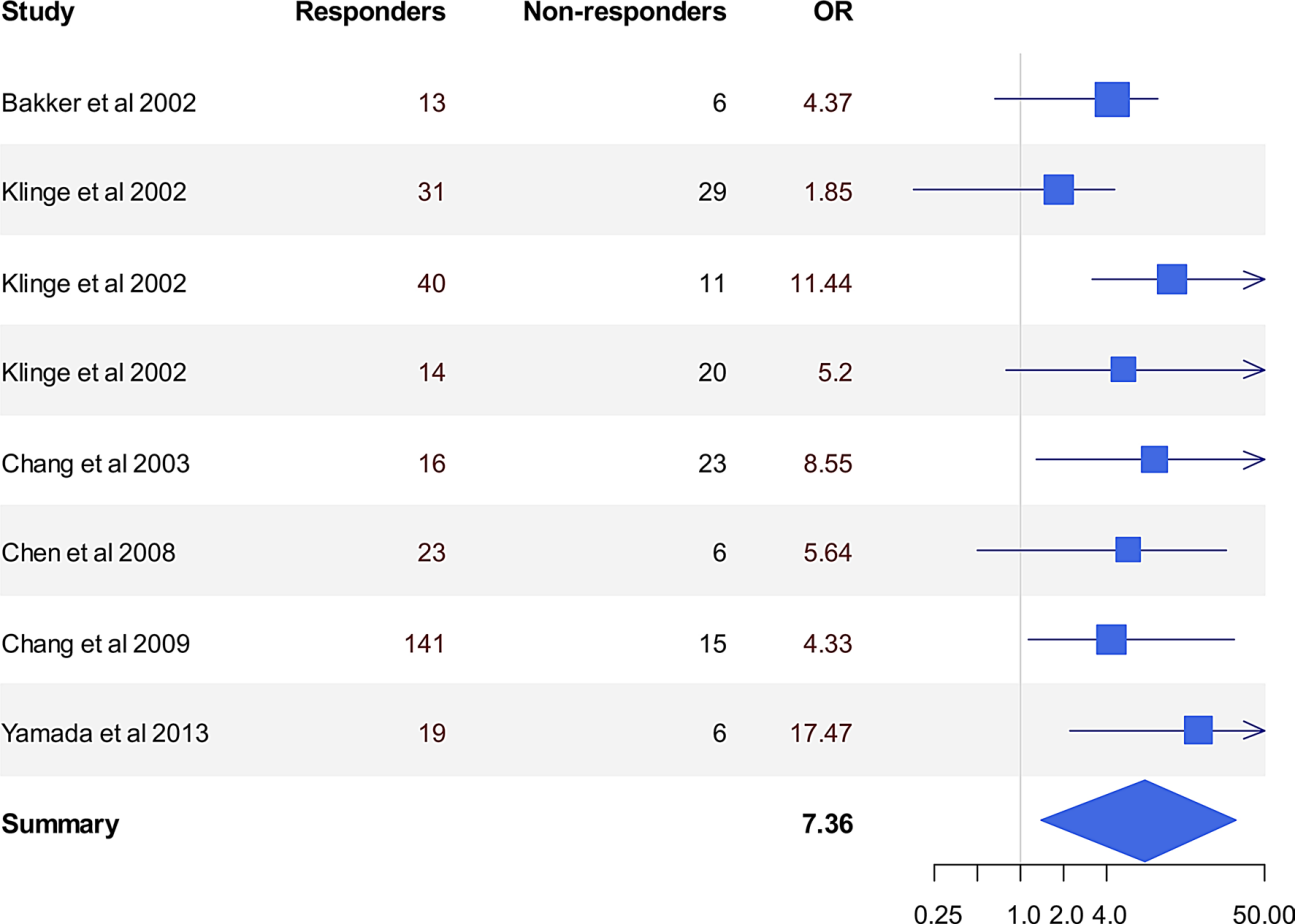
The predictive value of baseline cerebrovascular reactivity. The odds ratio (or) reflects the likelihood of baseline CVR to successfully differentiate between shunt responders and non-responders. All the studies except for Chen indicated that impaired preoperative CVR leads to a favourable outcome post-shunting

**Table 8:** Predictive value of baseline global and regional CBF for outcome after shunting

Reference	GRADE	Method	No of patients	Predictive Yes/No	Comments
**Global CBF**					
1. Mathew et al., 1975 +1977 [44]	**C**	Intracarotid 133Xe clearance during Angiography	**21**	**Yes**	Higher gCBF related to better clinical outcome
2. Hayashi et al., 1984 [49]	**B**	Intracarotid 133Xe clearance during Angiography	**16**	**Yes**	**Poor outcome if reduced gCBF <25** ml/100 g/min
3. Tanaka et al., 1997 [63]	**C**	**Xe contrast CT**	**21**	**Yes**	**Improvement with gCBF >20** ml/100 g/min
4. Klinge et al., 1998 [64]	**B**	H2O15 bolus PET	**21**	**Yes**	**Improvement with lower gCBF** 33 vs. 45 ml/1 00 ml per min responders vs non-responders
5. Klinge et al., 1999 [65]	**B**	H2O15 bolus PET	**10**	**Yes**	**Improvement with lower gCBF** Global. 36 vs 44 ml/100 ml/min responders vs non-responders
6. Bateman et al (2007) [67]	C	MRI (1.5T) with flow sequences	32	**No**	**20 responders vs 12 non-responders**
**Global & Regional CBF**					
7. Grubb et al 1977 [46]	**C**	H2O15 PET Intracarotid	**5**	**No**	
8. Lying-Tunnel [47]	**C**	AV-Difference NO2	**7**	**No**	
9. Graff-Radford et al., 1987 +1989 [24, 36]	**B**	**133Xe inhalation** **SPECT**	**56**	**Yes**	Improvement with increased Anterior/Posterior rCBF
10. Kushner et al 1984 [50]	**C**	As in 1.	**19**	**No**	
11. Mamo et al 1987 [55]	**B**	133Xe IV	**25**	**No**	
12. Meixensberger et al., 1989 [57]	**C**	**133Xe inhalation**	**31**	**No**	
13. Granado et al., 1991 [191]	**B**	**99m Tc-HMPAO** **SPECT**	**14**	**Yes**	**Improvement with increased Anterior/Posterior rCBF**
Regional CBF					
14. Morretti et al., 1988 [192]	**C**	123BAMP IV SPECT	**12**	**No**	Cortical rCBF, PVWM and basal ganglia examined
15. Ishii et al (2011) [80]	A	IMP SPECT rCBF relative to global brain activity.	84	**Yes**	In non-responders, 3 different patterns of reduction in rCBF identified (anterior, posterior and mixed)

**Table 9:** Baseline cerebrovascular reactivity and autoregulation: predictive value

Ref	GRADE	Method	No of patients	CVR predictive	Comments
1. Meyer et al., 1984 [51]	**C**	**CO2**	**7**	**Yes**	**Impaired** CVR associated with better outcome
2. Shimoda et al 1994 [150]	**C**	**glycerol**	**22**	**Yes**	More widespread increases in CBF post glycerol in shunt responders (**preserved** CVR).
3. Lee et al., 1998 [160]	**C**	**CO2**	**11**	**No**	
4. Klinge et al., 1999, 2002a, 2002b **(patient series)** [65, 162, 171]	**B**	^15^O-H_2_O PET; ACZ Low and high vascular risk factor groups	**53**	Yes in low risk group and no in high risk group	Baseline CVR was lower in responders than in non-responders (NS). Increase in CVR at 7d after shunting was predictive of a good outcome.
5. Chang et al (2003) [193]	B	**Tc**^**99m**^HCMP & ACZ	48	Yes	**↓ CVR** in 30 shunt responders compared to normals. 4 non-responders with preserved CVR.
6. Chen et al 2008) [194]	C	Xe-CT, MRI and MRSI	28	Yes	**Preserved** CVR ↓ improved .23 responders vs 5 non-responders
7. Chang et al (2009) [175]	B	SPECT and Xe-CT	162	Yes	**Impaired CVR** predictive. Responders w incomplete triad: **→preop CVR** than controls. Full triad:↓ preop CVR than in incomplete.
8. Yamada et al (2013)* [69]	B	^99m^Tc-ECD SPECT +ACZ	25	**Yes**	MMSE as improvement marker. <20% ↑ in preoperative CBF response to ACZ predicted improvement (**impaired**)
**Global Autoregulation**					
9. Lalou et al (2018) [170]	B	CSF infusion test (PRx) & non-invasive MAP	131	There was a trend towards higher values for PRx (greater global AR derangement) in non-responders v responders.	The product MAPx(1+PRx) – a measure of combined arterial hypertension and deranged AR, showed a significant association with outcome.

In contrast, studies of CVR are more promising. Although there is some inconsistency between studies, overall, it appears that impaired CVR in NPH patients was a favourable prognostic factor. 4 studies report that worse rather than good reactivity to ACZ predicts a favourable outcome [69, 151, 162]. One of them [151] interestingly stratified the patients into different groups depending on cardiovascular burden: worse reactivity predicted shunt responsiveness for high cardiovascular risk; better reactivity was related to shunt responsiveness in those with low cardiovascular risk. This interesting finding again highlights the importance of consideration of the degree of cerebrovascular disease in iNPH. 5 out of 5 studies that measured CVR post-operatively agree that it is significantly increased compared to pre-operatively and that restoration of CVR is a hallmark of shunt responsiveness [171]. Early detection of a CVR/CA problem may precede falls in global and rCBF, and white matter hyperintensities unlike in CSVD.

There has been only one study of global AR in relation to outcome after shunting [170]. There was a trend towards higher values for PRx (greater global AR derangement) in non-responders v responders associated with higher MAP values. The product MAP x (1+PRx) was proposed as a measure that combined arterial hypertension with deranged AR – this measure revealed a significant association with outcome.

### Relationship between CBF, CSF dynamics and the mechanical properties of the cerebral mantle

There is considerable evidence that the normal interrelationships between the cerebral circulation, the circulation of CSF and interstitial fluid and the mechanical properties of the cerebral mantle are altered in NPH.

#### Ventricular reflux and convexity block

NPH is characterised by ventricular reflux of CSF and convexity block [1, 2, 137, 195]. Intrathecal gadolinium tracer MR studies have confirmed these earlier findings with reduced clearance of gadobutrol and delayed tracer distribution over the external brain surface arteries compared with reference subjects [196]. The combination of DESH (Disproportionately enlarged subarachnoid space hydrocephalus) and FES (focally enlarged sulci) may prove to be a surrogate marker for convexity block. It has recently been suggested that focally enlarged sulci are not the result of atrophy but of CSF entrapment that reduce in size after shunting [197, 198]. Experimentally, convexity block can induce ventriculomegaly [199]. In NPH, ventricular reflux of CSF and convexity block is accompanied by increased MR mean diffusivity within the white matter indicative of raised extracellular water content manifesting as periventricular lucencies (PVLs), particularly around the frontal and occipital horns [200–202].

However, ventricular reflux and convexity block may occur in a minority of normal people and other conditions without hydrocephalus [203]. Findings of an abnormal CSF circulation on cisternography correlate to some degree with shunt responsiveness in NPH but neither are robust standalone predictors of outcome.

Hence, other factors apart from partial or complete reversal of the CSF circulation must be at play, including changes in brain biomechanics, to explain why an individual’s ventriculomegaly progresses and becomes symptomatic. As described previously, ventricular size in healthy people increases very rapidly after the age of 50 years [5, 6, 9, 10].

Longitudinal studies confirm that there is a presymptomatic stage to NPH when ventriculomegaly is present [5–8].

Graff-Radford and colleagues [36, 37] have suggested that some patients with NPH have head circumferences greater than in controls and suggested that the cause of the NPH was arrested congenital hydrocephalus becoming symptomatic later in life. Many patients with NPH have intracranial volumes significantly larger than normal, suggesting that the initial insult occurs before the sutures fuse at 1 year of age. Bradley et al [34, 35] also suggested that NPH may be a ‘two hit’ disease: benign external hydrocephalus in infancy followed by deep white matter ischaemia in late adulthood (see below).

#### Ageing, hypertension and CSVD

As noted earlier, CBF and vascular density in the brain decreases and the stiffness of the large elastic arteries increases with normal ageing. These ageing changes are compounded by chronic hypertension which induces remodelling and stenosis of the arteries and fibrinoid necrosis of the arterioles, attenuates cerebrovascular reactivity and functional hyperaemia, and shifts autoregulation to the right, thereby increasing vulnerability to hypotension and chronic, borderline ischaemia [204–210]. The human periventricular arterial blood supply is at particular risk [75, 211]. The increase in arterial stiffness is associated with elevated pulse pressure and blood flow pulsatility in the cerebral vasculature thereby releasing more pulsatile energy into the brain and leading to white matter hyperintensities and tissue damage which is associated with cerebrovascular/cognitive impairments. It is likely that NPH patients will be susceptible to episodes of reduced cerebral perfusion pressure particularly when cardiovascular disease, both systemic and CSVD, is present and autoregulation is impaired.

As described earlier, many NPH patients have both systemic hypertension and white matter vascular disease [23–30]. In Earnest’s original cases [23], autopsy showed extensive hypertensive cerebrovascular disease with multiple small infarcts of the deep cerebral and cerebellar grey and white matter but normal leptomeninges and arachnoid villi. One case improved with a shunt. Earnest proposed that hypertensive cerebrovascular disease, by causing multiple infarcts in the periventricular white matter and basal ganglia, may reduce periventricular tissue tensile strength and elastic properties permitting the ventricles to enlarge under the stress of the intraventricular pulse pressure when increased by hypertension. Some evidence for this concept has come from magnetic resonance elastography studies but the results are not consistent [212–217]. One confounding factor is the change in CSF outflow resistance and mechanical properties of the cerebral mantle as an individual’s NPH evolves [172, 218].

Interestingly, SHR rats, but not the more hypertensive SHRSP rats, develop chronic hydrocephalus with ventricular reflux [219–221]. The ventricular enlargement in SHRs does not develop as a direct consequence of the concomitant elevation in blood pressure - pharmacological blood pressure reduction failed to attenuate the ventricular enlargement in SHRs and experimentally induced hypertension was insufficient to cause ventricular enlargement in naïve rats. A meticulous study in the SHR rat by Macaulay’s group [173] has revealed that, although brain water does increase with ventriculomegaly, neither CSF production rate, ICP, nor CSF outflow resistance appear to be elevated when compared to WKY rats. They concluded that ‘SHR hydrocephalus represents a type of hydrocephalus that is not life threatening and occurs by unknown disturbances to the CSF dynamics’. Both subcortical (eg increased ventricular volume) and cortical (eg thinning and sulcal widening) atrophy can result from CSVD and Cadasil in the human [222–225]. Hence, although CSVD is very common in NPH, CSVD alone does not appear to be sufficient to induce NPH as opposed to atrophy.

### Compliance, resistance to CSF outflow and cerebral autoregulation

Global intracranial compliance is reduced in NPH despite, by definition, normal CSF pressure (mean 9–12 mm Hg). Pulsatile ICP, reflecting impairment of pressure-volume reserve capacity, increases as intracranial compliance is reduced [226]. Lundberg’s B waves occur more frequently and are increased in amplitude during sleep in NPH [227, 228]. However, such rises in ICP may not in general indicate ischaemia but are synchronised with rhythmical increases in cerebral blood volume [216, 229, 230]. As highlighted above, it has been suggested that such reduced intracranial compliance and increased intracranial pulse pressure might lead to local ‘‘barotrauma’’ or ‘‘tangential shear stress’’ [185–187, 231–234]. Increased intracranial and microvascular pulsatility might increase pulsatile stress forces and activate potent vasoactive factors, damage the cerebral microcirculation and even cause cognitive decline in elderly individuals (“pulse-wave encephalopathy”). Leukoaraiosis might reflect an arteriosclerotic and/or resistive pulse wave encephalopathy in mild cognitive impairment. Intriguingly, the Umea group have shown that elderly subjects with high intracranial pulsatility display smaller brain volume and larger ventricles, supporting the notion that excessive cerebral arterial pulsatility harms the brain [174]. However, In healthy older adults, the expression of white matter lesions and enlarged perivascular spaces precedes increases in cerebral arterial PI - elevated PI may be a relatively late manifestation, rather than a risk factor, for CSVD [235]. Using MR flow-quantification technology, Bateman has shown that intracranial arterial and sagittal sinuspulsatility was increased in NPH whereas CSF pulse was reduced [236]. Elevated CSF flow pulsation through the cerebral aqueduct have been widely described in communicating hydrocephalus but has not proven to be widely accepted as a good predictor of outcome after shunting [67, 237, 238].

Moreover, such enhanced pulsatility does not extend, at least in the adult rat with induced communicating hydrocephalus, to the entire cerebral vasculature including the cortical capillaries. Rashid et al [239] concluded that, even in the presence of markedly elevated pulsatile CSF flow in the aqueduct, there was no concurrent increase in microvascular pulsatile flow. A shunt may help in NPH, not by greatly decreasing CSF pressure or/and its dynamics but by increasing both compliance and perfusion.

Global intracranial compliance should be distinguished from intracranial arterial compliance. In the majority of patients with iNPH, there was a low correlation between intracranial and vascular pressure pulsatility. During moderate rises in ICP (eg up to 40 mmHg during an infusion test),TCD mean blood flow velocity only changes marginally -around 7%, compared to much higher percentage of ICP rise. The pulsatility index on the other hand, rises significantly. This reflects changes in compliance of the cerebral arterial walls, with autoregulation efficient enough to keep CBF constant. However, the ability of arteries in the subarachnoid space to expand, including during autoregulatory responses to changes in cerebral perfusion pressure, may be restricted. when intracranial compliance is reduced [240, 241]. Additionally, CPP during infusion is reduced by around 9% [242, 243]. Unlike with flow velocity assessment during infusion test, PET-H2O studies have shown a reduction of CBF by 10%, more so in the deep white matter [75]. This reduction in CBF appears to be proportional to the observed reduction in CPP as both shown flow velocity and CPP measurements studied during infusion testing.

We could therefore postulate that vasodilation in the MCA partially compensates for the decrease in perfusion during infusion. However, TCD does not interrogate white matter, and therefore perfusion changes measured with H2O-PET will not be reflected in the changes measured with TCD.

The cerebral periarterial spaces are dilated in NPH compared with controls [244]. In rodents, arterial hypertension reduces both arterial wall pulsatility and periarterial tracer movement [173, 219, 245]. It has been proposed that impaired intracranial compliance restricts cerebral arterial pumping, which in turn hampers the driving forces of perivascular molecular transport. As noted above, intrathecal gadolinium tracer MR studies in NPH subjects have demonstrated reduced clearance of gadobutrol and delayed tracer distribution over the external brain surface arteries compared with reference subjects (‘impaired glymphatic tracer clearance’).

The study of CSF dynamics and pressure-volume compensation combined with assessment of CBF provides an opportunity to assess the state of autoregulation using a variety of techniques including TCD and MRI [246–249]. For example, TCD enables the study of changes in CBF and global autoregulation continuously over time including overnight and during an infusion study [249]. The shape of the TCD pulse waveform is able to differentiate between hydrocephalus and normal subjects [250].

Unfortunately, relevant measurements using MRI have been limited by the strong magnetic field which excludes using invasive ICP instrumentation but Unnerbäck et al [251] have overcome this problem for patients in neurointensive care. Hence MR studies equivalent to PET studies [124, 125, 238] to assess autoregulation are feasible. Despite such limitations, the global and local biomechanical response of the brain to changes in CSF pressure may be studied using MRI to map CBF – stress/strain relationships and phase contrast MRI to characterize the CNS elastance coefficient during CSF infusion studies. A significant association between the CNS elastance coefficient, frailty and age has been reported, results that were independent of CSF dynamics and not specific to NPH [22]. Unpublished data from a large series using PC MRI suggests that there is a negative correlation between CBF, Rout and the baseline pulse amplitude of the ICP pulse waveform.

Lastly, studies of global autoregulation in NPH during CSF infusion studies using both the PRx index derived from continuous blood pressure monitoring and TCD-derived autoregulation (Mx index), have confirmed that there is a negative correlation between Rout and autoregulation [240]. This negative relationship at first seems counterintuitive. It might simply reflect the likelihood that global measurements of autoregulation do not detect dysautoregulation restricted to the periventricular zone in uncomplicated NPH. A high Rout and preserved global autoregulation might indicate that there is salvageable tissue which might be helped with a shunt. In contrast, global dysautoregulation would indicate more global cerebrovascular disease and ischaemia that has not affected resistance to CSF outflow.

These links between CBF, CSF pressure-volume compensation, the CSF circulation and periarterial fluid flow are very interesting but require further study in patients.

### Stress, stretch, compression and intramantle pressure gradients

It has long been postulated that ventriculomegaly creates regions of stress within the cerebral mantle, and thereby the interplay between mechanical and vascular factors [1, 2, 76, 130, 145, 252], Such stresses result in stretch and compression of both vascular and neural tissue, changes that may mirror the symptoms of NPH. Such effects have been successfully modelled for neural tissue using poroelastic theory [118, 119].

Crucially, Earnest’s hypothesis paved the way for subsequent studies of the biomechanics of the cerebral mantle in communicating hydrocephalus. The concept of a transmantle pressure gradient including differential pulse pressures, however small, between ventricle and subarachnoid space to explain ventricular dilatation in communicating hydrocephalus has not been demonstrated [253]. However, finite element studies have demonstrated that both ventricular expansion induced by CSF infusion and brain deformation are accompanied by heterogeneous stress concentrations within the cerebral mantle [119, 120, 131, 238]. Mean stress, indicating compression, was distributed throughout the brain during CSF infusion and maximal in the thalamus and corpus callosum. The white matter surrounding the ventricular horns was the site of maximal shear stress. Subsequent to brain deformation, one would expect some distortion to local arterial trees and thus a compromise to rCBF. In an instructive case report, brain deformation caused by a cyst was accompanied by large shear stresses and moderate compressive stresses consistent with areas of hypoperfusion on PET rCBF maps and specific cognitive deficits [119]. Only small interstitial fluid pressure changes were present.

Ventricular dilatation has often been taken as evidence that the brain is being compressed and that an increase in intra-parenchymal pressure results [254]. However, there is no direct evidence to suggest that the intra-parenchymal pressure is increased, quite the reverse. Pena et al have proposed, based on a finite element analysis of a poroelastic model, that ventricular expansion may result from a relative reduction in interstitial fluid pressure in the periventricular area leading to the formation of a ventricle - parenchymal rather than ventricle – subarachnoid pressure gradient [255]. This concept has received experimental and modelling support by Johnston’s group [256, 257]. It will be fascinating to see what the impact of the recently described genetic risk variants for NPH have on paraventricular biomechanics and ISF flow, and whether they overlap with those for CSVD [258, 259].

#### Bias assessment

There was frequently a moderate to high risk of selection and prognostic factor measurement bias in the current evidence, mainly due to the absence of unified criteria that include comorbidities for defining and selecting the study participants. Furthermore, there are no randomized trials specifically designed to assess outcome in the current literature, utilising CBF as a prognostic factor.

#### Future directions

As noted in the 2001 systematic review, large multicentre platforms are required to provide the infrastructure for producing higher level evidence with reduced risk of bias based on a general consensus over definitions including clinical heterogeneity, inclusion/exclusion criteria, comorbidities including frailty, and outcome measures. Significant progress has been made in building such platforms as exemplified by the publication and adoption of International NPH Guidelines [260–262], multinational RCTs [263, 264], FinnGen Cohort [258, 259], Registries (UK, Sweden & Australasia [4, 11, 265] and NPH Special Interest Groups (eg Hydrocephalus Society, Association of British Neurologists UK).

The mechanism(s) governing normal autoregulation are complex [177, 178]. Similarly, the mechanisms underlying impaired cerebrovascular reactivity and autoregulation in NPH remain an enigma. Future studies should include mapping of regional variations in haemodynamic reserve onto intracerebal water content, state of myelination, resting state networks, CSF periarterial inflow and outflow pathways, vascular co-morbidity and CSVD.

A holy grail of NPH research is to identify accurate predictive biomarkers, alone or in combination, of outcome after shunting. The relationship between response to a tap test and outcome after shunting is capricious. The interpretation of CSF outflow resistance depends on age, duration of symptoms and co-morbidities. The higher the Rout, the greater the probability of a favourable outcome after a shunt but a minority of patients with a normal Rout still improve after shunting. Interestingly, cerebrovascular reactivity tests have predictive value. One possibility would be an RCT that explores the predictive accuracy of the combination of a tap test/EDLT with CVR.

Apart from shunting, there are no other proven treatments apart from conventional management of frailty, vascular risk factors and comorbidities including, for example, PD and CSVD. Blood pressure control may help to prevent white matter hyperintensities in CSVD [266]. ACZ was discovered in the 1940’s as an inhibitor of carbonic anhydrase. ACZ has well known effects on CSF production by the choroid plexus and as a cerebral vasodilator. In a pioneering French pilot study [267], ACZ was found to clinically improve 10 out of 15 NPH patients examined over 2 years. Tolerance was excellent with a daily dose of 250 to 500 mg. The benefit remained stable after 1 year follow-up in 8 cases. Low-dose ACZ was later shown to reverse white matter lesions in NPH within a few months [200, 268]. The results of the Uppsala DRAIN RCT are keenly awaited (EudraCT Number 2020-004132-22).

### Summary of major findings

- Overall, NPH patients have an average global reduction of CBF of 15%. Regional hypoperfusion in the periventricular white matter, basal ganglia and thalamus have been shown consistently. These regional patterns may be characteristic of NPH and directly related to ventriculomegaly and higher-level gait disorder. However, the remainder of signs and symptoms of NPH could not be consistently localised to relevant loci of hypoperfusion-hypometabolism.
- PET studies have confirmed that this mild global reduction in CBF was accompanied by reduction in cerebral glucose and oxygen consumption but with a modest increase in OEF in some but not all studies. No OEF data is available for periventricular grey and white matter. Intracerebral microdialysis studies provide some further evidence of borderline, chronic ischaemia in NPH.
- No global or regional CBF changes are predictive of outcome after CSF drainage or shunting. Therefore, there is very limited scope for those measurements in aiding clinical practice.
- Global and Regional cerebrovascular reactivity and autoregulation appear to be closely linked to the disturbed CSF circulation and resulting tissue distortion in NPH. Whilst preliminary studies on their predictive value have shown some promise, additional evidence is needed to further evaluate their predictive value.
- Based on the current literature, we would conc;ude that the cerebral mantle is partially protected from serious ischaemia by appropriately reduced metabolic demand and recruitment of the vasodilatory capacity of the cerebrovascular bed. Although there are no relevant longitudinal studies of cerebrovascular reserve prior to shunting, it is likely that, when this capacity is exhausted, irreversible tissue damage may ensue with autoregulatory loss, irreversible ischaemia and atrophy.
- In light of the limited prognostic value of current CBF assessments in NPH and the emerging significance of cerebrovascular reactivity as both a pathophysiologic marker and a potential predictor of treatment response, a trial integrating haemodynamic reserve, CSF outflow resistance, and temporary CSF drainage is indicated. Such a multidimensional approach could redefine our framework of knowledge and understanding, as well as unlock more precise therapeutic stratification for NPH patients.”

## Conclusions

Both baseline rCBF and borderline ischaemia approximate to the cortical and subcortical circuitry that contribute to the clinical features of NPH but are not predictive of the response to shunting. Impaired cerebrovascular reactivity has predictive value for the clinical response to shunting. In addition to RCTs of carbonic anhydrase inhibitors, consideration should be given to an RCT that explores the predictive accuracy of the combination of the clinical response to temporary CSF drainage with changes in CVR.

## Data Availability

No datasets were generated or analysed during the current study.

## Abbreviations

CBF: Cerebral Blood Flow
rCBF: Regional Cerebral Blood Flow
tCBF: Total Cerebral Blood Flow
CVR: Cerebrovascular reactivity
GRAD: Grading of Recommendations Assessment, Development, and Evaluation
(i)NPH: (idiopathic) Normal Pressure Hydrocephalus
RTI: From RTI international, no acronym clarification provided. https://www.rti.org/about-us
STT: Spinal Tap Test
TCD: Transcranial Doppler
